# Ex-Vivo ^13^C NMR Spectroscopy of Rodent Brain: TNF Restricts Neuronal Utilization of Astrocyte-Derived Metabolites

**DOI:** 10.1021/acs.jproteome.4c00035

**Published:** 2024-06-29

**Authors:** Daniel Radford-Smith, Tang T. Ng, Abi G. Yates, Isobel Dunstan, Timothy D. W. Claridge, Daniel C. Anthony, Fay Probert

**Affiliations:** † Department of Chemistry, University of Oxford, Oxford OX1 3TA, U.K.; ‡ Pharmacology Department, University of Oxford, Oxford OX1 3QT, U.K.

**Keywords:** ex-vivo NMR, stable isotope tracing, neuroinflammation, metabolomics, astrocyte, tumor necrosis factor

## Abstract

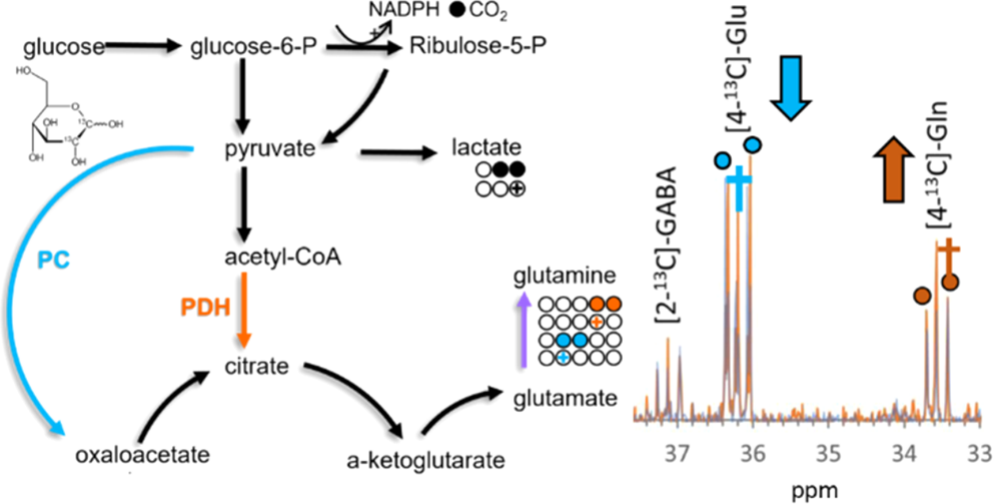

Tumor necrosis factor (TNF) has well-established roles in neuroinflammatory disorders, but the effect of TNF on the biochemistry of brain cells remains poorly understood. Here, we microinjected TNF into the brain to study its impact on glial and neuronal metabolism (glycolysis, pentose phosphate pathway, citric acid cycle, pyruvate dehydrogenase, and pyruvate carboxylase pathways) using ^13^C NMR spectroscopy on brain extracts following intravenous [1,2-^13^C]-glucose (to probe glia and neuron metabolism), [2-^13^C]-acetate (probing astrocyte-specific metabolites), or [3-^13^C]-lactate. An increase in [4,5-^13^C]-glutamine and [2,3-^13^C]-lactate coupled with a decrease in [4,5-^13^C]-glutamate was observed in the [1,2-^13^C]-glucose-infused animals treated with TNF. As glutamine is produced from glutamate by astrocyte-specific glutamine synthetase the increase in [4,5-^13^C]-glutamine reflects increased production of glutamine by astrocytes. This was confirmed by infusion with astrocyte substrate [2-^13^C]-acetate. As lactate is metabolized in the brain to produce glutamate, the simultaneous increase in [2,3-^13^C]-lactate and decrease in [4,5-^13^C]-glutamate suggests decreased lactate utilization, which was confirmed using [3-^13^C]-lactate as a metabolic precursor. These results suggest that TNF rearranges the metabolic network, disrupting the energy supply chain perturbing the glutamine-glutamate shuttle between astrocytes and the neurons. These insights pave the way for developing astrocyte-targeted therapeutic strategies aimed at modulating effects of TNF to restore metabolic homeostasis in neuroinflammatory disorders.

## Introduction

Stable isotope tracing combined with NMR spectroscopy is a powerful tool that can probe unique metabolite pathways that dominate specific cell types in whole tissue extracts *ex vivo*. The use of [1,2-^13^C]-glucose as a metabolic substrate has been shown to be particularly valuable in the study of glia and neuronal metabolic pathways by providing information on glycolysis, the pentose phosphate pathway (PPP), TCA cycle, glutamine/glutamate shuttle, pyruvate dehydrogenase (PDH), and pyruvate carboxylase (PC) metabolism in a single experiment.^1−4^ Here, we sought to utilize the power of stable isotope tracing NMR spectroscopy to investigate glial and neuronal metabolism simultaneously, following a challenge with Tumor necrosis factor α (TNF) in whole rodent brain extracts.

TNF is a proinflammatory cytokine that plays a central role in the development of neuroinflammatory diseases. While the development of anti-TNF therapy has undoubtedly revolutionized the treatment of diseases such as rheumatoid arthritis and inflammatory bowel disease,^5^ these therapies can exacerbate neuroinflammatory diseases of the central nervous system (CNS), such as multiple sclerosis.^6−8^ Despite its clear role in neuroinflammation, the precise effect of TNF on the cellular metabolism within the brain remains poorly understood. TNF is responsible for maintaining synaptic plasticity and is neuroprotective in the healthy brain. Conversely, in the inflamed brain, TNF has been shown to promote the generation of reactive astrocyte phenotypes that may contribute to neurotoxicity.^9^ Thus, it is essential to study the direct effects of TNF on glial (with a focus on astrocytes) and neuronal metabolic processes to better understand the role of this important cytokine in the development of neuroinflammatory diseases. We hypothesized that ex vivo ^13^C NMR of rodent brain extracts infused with [1,2-^13^C]-glucose, [2-^13^C]-acetate, or [3-^13^C]-lactate as the metabolic precursor could provide novel information on the metabolic interplay between astrocytes and neurons following a TNF challenge.

**Figure 1:**
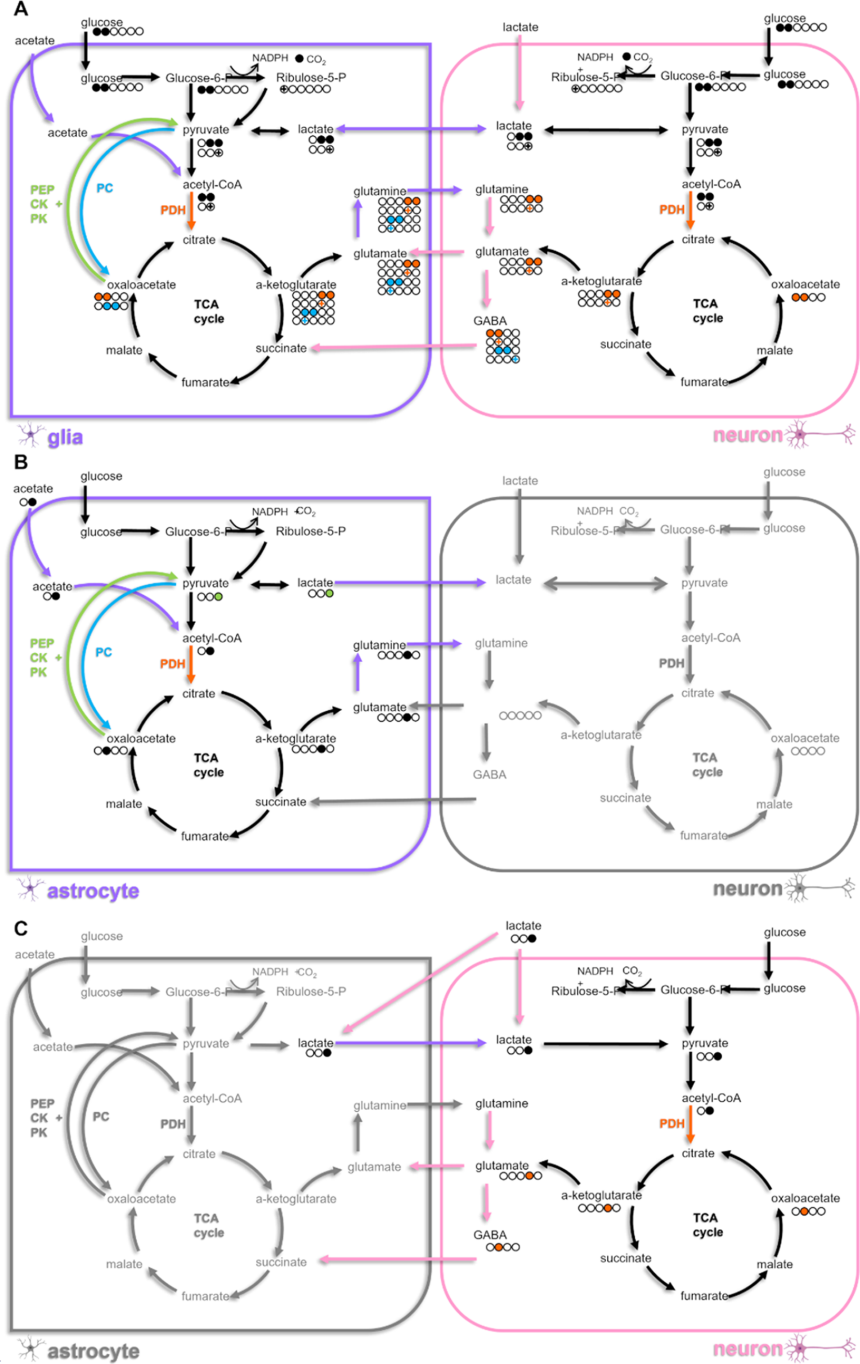
Schematic illustrating labeling of key astrocytic (purple) and neuronal (pink) metabolites following metabolism of (A) [1,2–13C]-glucose, (B) [2–13C]-acetate, and (C) [3–13C]-lactate. The carbon backbone of each molecule is represented by circles. Labels resulting from glycolysis are represented by solid fills, while PPP metabolism is represented by crosses (+). Labels resulting from metabolism via both PDH (orange), PC (blue), and pyruvate recycling (green) pathways are represented. Labeling resulting from multiple turns of the TCA cycle and back-flux are omitted to preserve clarity. Glucose-6-P; glucose-6-phosphate, NADPH; nicotinamide adenine dinucleotide phosphate, PDH; pyruvate dehydrogenase, PC; pyruvate carboxylase, PEPCK; phosphoenolpyruvate carboxykinase, PK; pyruvate kinase.

The use of a [1,2-^13^C]-glucose substrate provides a substantial amount of information, allowing glial and neuronal metabolism simultaneously, while also distinguishing between glycolytic and PPP metabolites. Metabolites downstream of either glycolysis or the PPP are readily identified by inspection of the ^13^C NMR splitting pattern; this is a distinct advantage of *ex vivo* NMR methods relative to lower resolution *in vivo* analysis methods. Any downstream metabolites resulting from glycolysis of [1,2-^13^C]-glucose will retain both ^13^C atoms, resulting in doublet peaks. In contrast, during PPP, oxidative decarboxylation of glyceraldehyde-6-phosphate (G6P) generates NADPH and ^13^CO_2_. Thus, any downstream metabolites contain a single ^13^C atom, resulting in a singlet peak (Figure 1A). While the presence of singlet or doublet peaks in the ^13^C NMR spectrum allows metabolites arising via glycolysis or PPP to be easily identified, inspection of the position of the ^13^C label in metabolites downstream of the TCA cycle, particularly glutamine and glutamate, allows for astrocytic and neuronal pathways to be distinguished.

Glutamine and glutamate metabolism is highly compartmentalized within the brain with glutamate production via neuronal glutaminase shuttled to astrocytes for conversion to glutamine for transport back to the neuron as part of the glutamine-glutamate cycle. While glutaminase is expressed in low concentrations in astrocytes, it is considered a neuronal maker. In contrast, neurons lack the capacity to synthesize glutamine themselves. Thus, the measurement of total glutamine and glutamate concentrations can be considered to be markers of astrocytic and neuronal metabolism, respectively.

In neurons, glycolysis of [1,2-^13^C]-glucose results in [2,3-^13^C]-pyruvate, which is reduced to [1,2-^13^C]-acetyl CoA by PDH. Aldol condensation of [1,2-^13^C]-acetyl CoA with oxaloacetate forms [4,5-^13^C]-citrate, which is isomerized and oxidized to α-[4,5-^13^C]-ketoglutarate in the TCA cycle. α-[4,5-^13^C]-ketoglutarate is a precursor of [4,5-^13^C]-glutamate, which is then shuttled to the astrocyte.^10^

In astrocytes, [4,5-^13^C]-glutamate is transaminated to [4,5-^13^C]-glutamine by glutamine synthetase (GS) before being shuttled back to the neuron.^11,12^ [2,3-^13^C]-pyruvate can also be metabolized by PC, an astrocyte-specific enzyme,^13^ leading to [2,3-^13^C]-oxaloacetate and [3,4-^13^C]-citrate. Isomerism and oxidation of [3,4-^13^C]-citrate gives rise to α-[2,3-^13^C]-ketoglutarate, [2,3-^13^C]-glutamate, and [2,3-^13^C]-glutamine. Thus, labeling patterns specific to the PC pathway (namely, [2,3-^13^C]-glutamine) indicate alterations in glial metabolism, while changes in [4,5-^13^C]-glutamate (produced via PDH) provide insight into neuronal metabolism (although contributions from astrocytic metabolism cannot be ruled out using the [1,2-^13^C]-glucose substrate alone).

The results from the use of [1,2-^13^C]-glucose as a metabolite precursor can be further validated using alternative substrates preferentially metabolized by specific cell types. Acetate^14,15^ is considered an astrocyte-specific substrate, and so its combination with [1,2-^13^C]-glucose can be used to identify the astrocyte contribution to the metabolic changes identified. [2-^13^C]-acetate is combined with coenzyme A (CoA) by acetyl CoA synthetase to form [2-^13^C]-acetyl CoA which undergoes a condensation reaction with oxaloacetate, resulting in [4-^13^C]-citrate and correspondingly labeled [4-^13^C]-glutamate and [4-^13^C]-glutamine. [3-^13^C]-lactate can be oxidized to [3-^13^C]-pyruvate by lactate dehydrogenase. Reduction of [3-^13^C]-pyruvate gives rise to [2-^13^C]-acetyl CoA, and the subsequent single C4 label in citrate, α-ketoglutarate, glutamate, and glutamine (Figure 1B).

While the classical hypothesis of the astrocyte-neuron lactate shuttle suggests that lactate (produced by astrocytes) is preferentially utilized by neurons^16−18^ to produce glutamate (Figure 1C), the precise conditions under which this occurs remain debated and so contributions from astrocytic glutamate synthesis cannot be ruled out in [3-^13^C]-lactate infused samples. Nevertheless, [3-^13^C]-lactate can be a useful metabolic precursor to probe glutamate synthesis in further detail.

Here we demonstrate that ^13^C NMR of brain extracts from rodents infused intravenously with [1,2-^13^C]-glucose provides novel information about the dysregulation of glial and neuron metabolism as a result of TNF, in a single experiment. Notably, an increase in glutamine and decrease in glutamate as a result of TNF treatment points to dysregulation of the glutamine-glutamate shuttle. The contribution of astrocytes to the observed changes in glutamine is confirmed using [2-^13^C]-acetate as a metabolic precursor, while treatment with [3-^13^C]-lactate confirms a reduced capacity of the brain to synthesize glutamate from this alternative energy source.

## Experimental Section

### Animals

Wistar rats (76–100 g), purchased from Charles River UK, were housed under a 12 h light/dark cycle with *ad libitum* access to food and water. All procedures were carried out in accordance with the UK Animals (Scientific Procedures) Act, 1986, and licensed protocols were approved by local committees (LERP and ACER, University of Oxford) and carried out under the UK Home Office license P996B4A4E.

For all intracranial injections, animals were anesthetized using 2.8% isoflurane and were mounted within a stereotactic frame.^19^ A midline scalp incision was performed to expose the skull, and a burr hole was drilled followed by intracranial (IC) injections administered via finely drawn glass capillaries (tip Ø < 50um) into the left striatum (From bregma: A/P −1.0 mm, M/L −3.0 mm, D/V −4.0 mm). Postinjection, the skin was sutured, and the rats were kept in a heated chamber (37 °C) until they regained consciousness, after which they were returned to their home cage.

To investigate the effect of TNF on brain metabolism with different ^13^C-labeled substrates, rats received 1 μL, administered over 5 min, as intracranial injections of either vehicle alone (0.1% BSA in 0.9% NaCl) as control or 1.5 μg.μL^–1^ recombinant rat TNF (R&D systems) diluted in the vehicle. After 24 h, rats received an intravenous infusion, over 10 min under isoflurane (*n* = 6/group), of 1 mL of sterile saline containing 1.1 mM [1,2-^13^C]-glucose, or [2-^13^C]-acetate, or [3-^13^C]-lactate. Animals were culled 30 min after initiation of the infusion by cardiac puncture under isoflurane before immediate dissection. For L-lactate treatment experiments, animals received an intraperitoneal injection of L-lactate in sterile saline (1g/kg body weight), or vehicle solution, 5 min after TNF administration and repeated 24 h later, immediately prior to infusion with [1,2-^13^C]-glucose. Animals were then transcardially perfused with cold heparinized saline, and the fresh brain tissue was collected, immediately frozen in isopentane on dry ice and stored at −80 °C for later analysis. Notably, this infusion strategy resulted in increased ^13^C enrichment (above natural abundance) by between 47 and 170% (Figure S1).

In the study, the dissection method involved the complete removal of the entire brain, followed by precise full coronal slice incisions made 1.5 mm anterior and posterior on either side of the injection site on both the same side (ipsilateral) and the opposite side (contralateral) as the injection, as well as along the longitudinal fissure to separate each hemisphere. Tissue was immediately frozen and stored at −80 °C until further analysis.

### Histology

Brain tissue was stained for glial fibrillary acidic protein (GFAP) [**G4546, Sigma-Aldrich]**. All steps were carried out at room temperature unless otherwise stated. Sections were rehydrated in PBS and postfixed in 4% neutral buffered formaldehyde (Sigma-Aldrich) for 10 min, followed by antigen retrieval with citrate buffer (pH 6.0, 95 °C) for 15 min to unmask GFAP antigens. Tissue sections were blocked with 10% goat serum in PBS for 1 h to prevent nonspecific binding. The sections were immunostained overnight at 4 °C with a rabbit antirat GFAP (Sigma-Aldrich, G4546, 1:1000) in 1% goat serum in PBS. Sections were then washed in PBS and incubated with biotinylated goat antirabbit (Vector Laboratories, 1:200) secondary antibody for 2 h, followed by avidin–biotinylated enzyme complex (Vectastain Elite ABC Standard, 1:100, Vector Laboratories) for 1 h. Staining was visualized with 125 μL H_2_O_2_ solution and 3,3′-diaminobenzidine (DAB) in 300 mL of phosphate buffer. Sections were counterstained with cresyl violet, gradually dehydrated through an increasing gradient of ethanol concentrations (80%, 95%, twice with separate 100% solutions), and cleared in xylene. Finally, the sections were mounted using DPX mounting medium (Fisher).

### Image Processing

Images of the full striatum (intracranial injection site) per hemisphere were acquired using a Nikon Labophot-2 microscope, followed by image analysis using ImageJ2, v214.0/1.54f, and the DAB plugin. Five nonoverlapping, 250 μm^2^ fields with the most intense GFAP staining within the striatum were selected, and the area of GFAP-positive staining was calculated for each hemisphere. Values were normalized by dividing the ipsilateral values by the contralateral values from the same animal.

### Metabolite Extraction

The brain tissue was weighed and mechanically homogenized 12.5% w/v in cold 50% v/v acetonitrile in ddH_2_O. The mixture was vortexed followed by centrifugation (5060*g* for 5 min at 4 °C). A constant volume of supernatant across all samples, determined by the volume corresponding to the lowest tissue mass, was aspirated to a new Eppendorf tube. The extraction was repeated, and the second supernatant was added to the initial aspirated supernatant. The pooled fractions were frozen on dry ice before being lyophilized overnight. The freeze-dried samples were stored at −80 °C until NMR analysis. To maintain metabolite integrity, all extraction steps were performed on dry ice with all samples processed in parallel, within 30 min.

### NMR Sample Preparation

The frozen brain lyophilized extracts were reconstituted in 600 μL of 75 mM sodium phosphate buffer D_2_O (pH 7.4) containing 33.8 μM 3-(trimethylsilyl)propionic-2,2,3,3-d_4_ acid (TSP) as an NMR reference (Sigma-Aldrich). Resuspended samples were gently mixed and clarified by centrifugation (2500*g* for 5 min at 4 °C) to remove particulates. The supernatant was transferred to a 5 mm NMR tube.

### NMR Acquisition

All ^1^H NMR experiments were carried out on a 700 MHz Bruker AVIII spectrometer operating at 16.4 T equipped with an inverse ^1^H/^13^C (^15^N) TCI cryoprobe at 298 K (Department of Chemistry, University of Oxford). ^1^H NMR spectra were acquired using a 1D NOESY presaturation pulse sequence to attenuate the large water signal with a 2 s irradiation and 8 or 32 scans for plasma samples and brain samples, respectively. Proton-decoupled ^13^C NMR spectra were obtained by using a standard 30° excitation pulse and pore-gated ^1^H decoupling with 2048 scans and a relaxation decay of 2 s.

### Data Preprocessing

Resulting free induction decays (FIDs) were multiplied by an exponential function corresponding to 0.3 Hz line broadening prior to Fourier transformation in TopSpin 4.1.4 (Bruker, 2022). The spectra were phased, baseline corrected, and referenced to the lactate-CH_3_ doublet resonance at δ = 1.33 or 22.8 ppm for ^1^H NMR and ^13^C NMR, respectively. Spectra were visually examined for poor baseline correction, spectral distortion, referencing errors, or contamination. Regions corresponding to noise, residual water resonances, or contaminants were removed from further analysis, and the remaining spectral regions were split into buckets of 0.02 ppm for ^1^H spectra or manually selected in ^13^C spectra in ACD/Laboratories Spectrus Processor Academic Edition 12.01 (Advanced Chemistry Development, Inc., 2010). The integral of each spectral bucket was calculated and normalized to the sum of the total spectral integrals. Isolate the effect of TNF treatment from other sources of variation (including biological variation and variation as a result of sample handling), and ipsilateral (left hemisphere, treated with either TNF or vehicle) values were divided by the contralateral (right hemisphere, untreated) values that acted as internal controls, reported as ipsilateral/contralateral hemisphere ratios. Metabolites were assigned using literature values,^20−22^ the Human Metabolome Database,^23^ and via 1D and 2D total total correlation spectroscopy (TOCSY) spectra.

### Statistical Analysis

All statistical analysis was carried out using in-house R scripts and the *ropls* package in RStudio. Principal component analysis (PCA) was applied to the normalized integral values to visualize separation between different types of intracranial injection. The buckets with the largest loadings, and hence the greatest variance, were identified, and the metabolites in these regions were assigned. Ellipses plotted on PCA score plots represent Hotelling’s 0.95 significance level.

Univariate analysis was applied to the metabolites of interest using either a two-tailed Student’s *t* test or a two-way analysis of variance (ANOVA) and posthoc Tukey’s multiple comparisons test. *P*-values less than 0.05 were considered significant. All *p*-values reported follow a Bonferroni multiple comparison correction.

## Results & Discussion

### TNF Induces Profound Changes in the Global (^1^H NMR) Brain Metabolome

**Figure 2:**
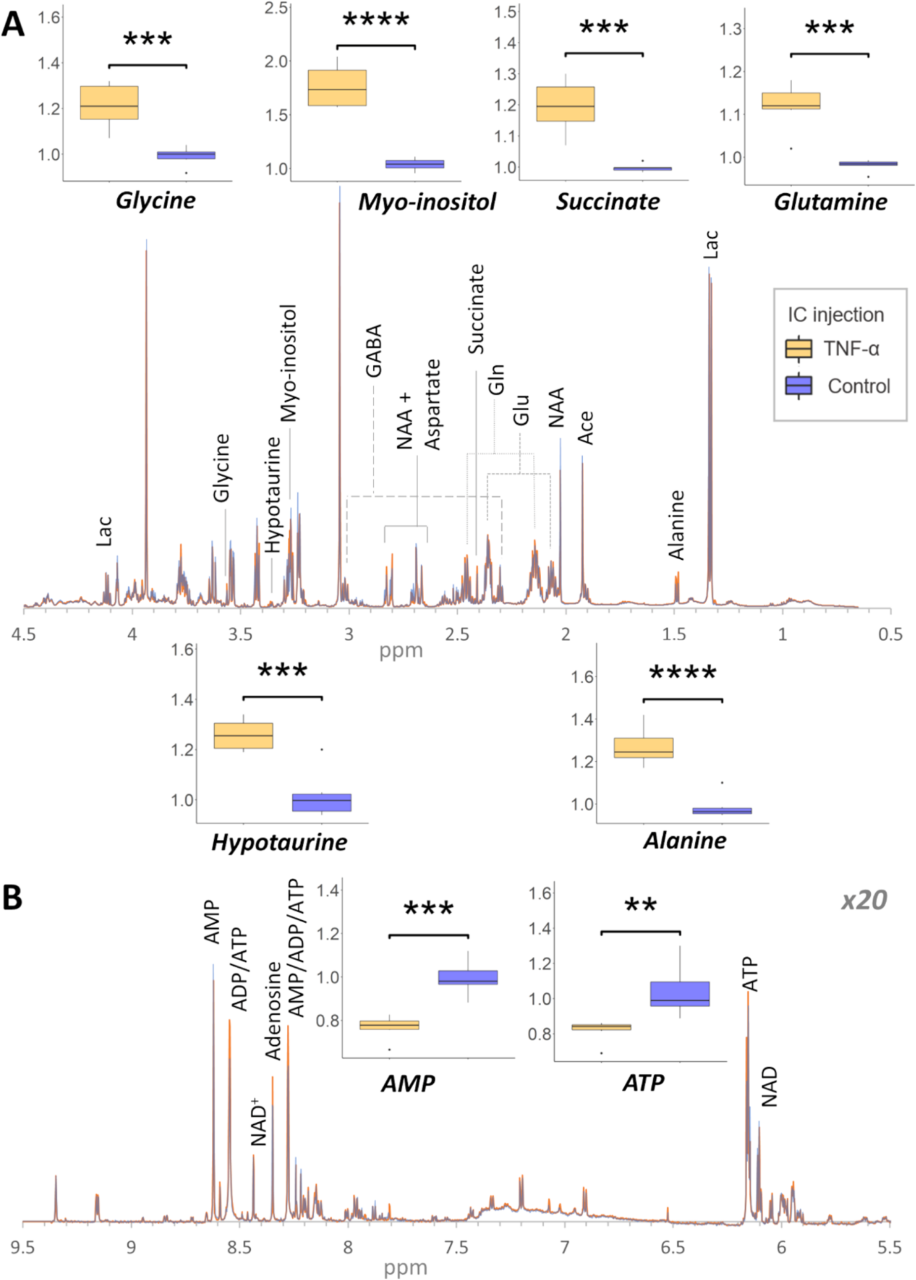
Mean 700 MHz 1H NMR spectra of the ipsilateral brain extracts obtained from the vehicle-treated (blue, *n* = 6) and TNF-treated (orange, *n* = 6) rats with significant metabolites labeled. Box plots show the significance of discriminatory metabolites identified from PCA loadings. A. 0.5–4.5 ppm spectral region. B. 5.5–9.5 ppm spectral region with the resonance intensity amplitude increased by 20-fold relative to A. IC, intracranial; Ace, acetate; AMP/ADP/ATP, adenosine mono/di/tri- phosphate; GABA, γ-aminobutyric acid; Gln, glutamine; Glu, glutamate; Lac, lactate; NAA, N-acetyl aspartate; NAD, nicotinamide adenine dinucleotide. Student’s *t* test *p*-values less than 0.05, 0.01, 0.001, and 0.0001 are represented by *, **, ***, and ****, respectively.

The ^1^H NMR spectrum provides a measure of total metabolite concentrations (irrespective of ^13^C label incorporation), providing information about the global metabolome. The ^1^H NMR profiles of brain extracts from rats treated with either TNF or vehicle (controls) were readily separated by PCA, confirming that TNF has a profound impact on the brain metabolome as a whole (Figure S2). Interrogation of the loadings was performed (Figure S4) followed by univariate analysis revealed that the concentrations of alanine, glutamine, glycine, hypotaurine, myo-inositol, and succinate were significantly increased, while the concentration of adenosine monophosphate (AMP) and adenosine triphosphate (ATP) was significantly decreased in TNF relative to controls (Figure 2). It should be noted that the ipsilateral/contralateral hemisphere ratio of ^1^H NMR-detectable metabolites ranged from 0.96 to 1.04 in the vehicle-treated group, confirming that the intracranial injection itself had a minimal impact on the ^1^H brain metabolome. Furthermore, immunohistochemistry revealed a significant decrease in GFAP staining in TNF-treated samples consistent with a TNF-induced reduction in intracellular GFAP as previously described^24−26^ (Figure S2). A full list of ^1^H assignments can be found in the Supporting Information (Table S1).

The significant increase in total glutamine concentration following TNF is likely a result of increased glutamine production due to activation of reactive astrocytes.^27^ Interestingly, no significant difference was observed in glutamate concentration as a result of TNF, suggesting a block in the glutamine/glutamate shuttle. The TNF-induced activation of astrocytes also results in a marked increase of T-cell and monocyte infiltration in the brain.^28,29^ It has been shown *in vitro* that naïve T cells depend on exogenous alanine for activation,^30^ thus the observed increase in alanine may suggest the activation of T cells while the increase in glycine and hypotaurine may be a result of ameliorating responses to reactive oxygen species induced by TNF.^31−33^ Interestingly, no significant difference in taurine concentration was observed despite its higher prevalence in the brain than hypotaurine. Myo-inositol on the other hand is an astrocytic osmolyte, regulating water content,^34^ which may increase in response to increased expression of AQP4 and water influx in astrocytes induced by TNF.^35,36^

### [1,2-^13^C]-Glucose, [2-^13^C]-Acetate, and [3-^13^C]-Lactate Have No Significant Impact on the Global (^1^H) Brain Metabolome

**Figure 3:**
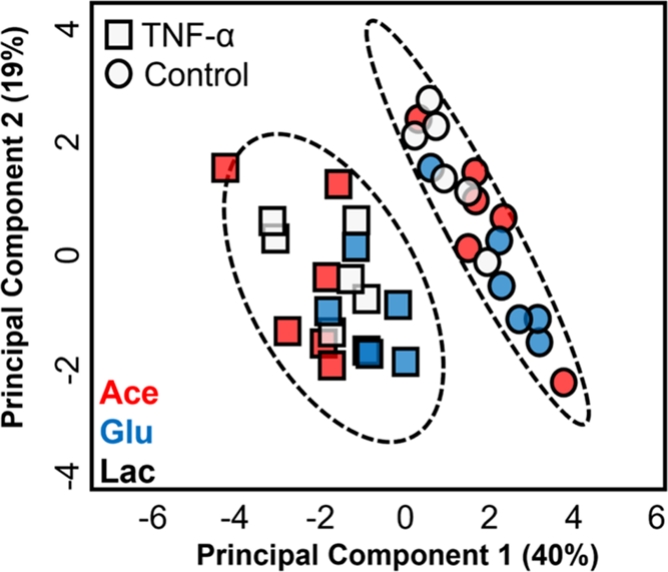
PCA scores plot (R2 = 0.584) showing separation between TNF (squares) and vehicle-treated control (circles) groups following intravenous infusion with [2–13C]-acetate (red), [1,2–13C]-glucose (blue), or [3–13C]-lactate (gray).

To confirm that infusion of ^13^C metabolite substrates themselves has no significant impact on the brain metabolome and does not confound the effect of TNF, we next compared the ^1^H brain metabolite profiles from rats infused with either [1,2-^13^C]-glucose, [2-^13^C]-acetate, or [3-^13^C]-lactate with and without TNF treatment. The PCA scores plot clearly demonstrates that infusion of ^13^C substrates has no appreciable impact on the brain metabolome of the control (vehicle-treated) animals while the significant perturbations as a result of TNF treatment remain (Figure 3). Furthermore, the significant metabolite changes (selected by inspection of the PCA loadings [Figure S4]) identified following TNF treatment were reproduced in each of the ^13^C substrate-infused groups, with the exception of ATP in the ^13^C-lactate infused group, which, while showing a decreasing trend similar to all other groups, did not reach significance (Figure S5). Thus, these results suggest that the choice of metabolic substrate does not significantly alter the ^1^H metabolome or confound our results.

### TNF Promotes Glycolytic Flux and Upregulates Glutamine Production by Astrocytes While Simultaneously Ssuppressing Glutamate Synthesis

**Figure 4:**
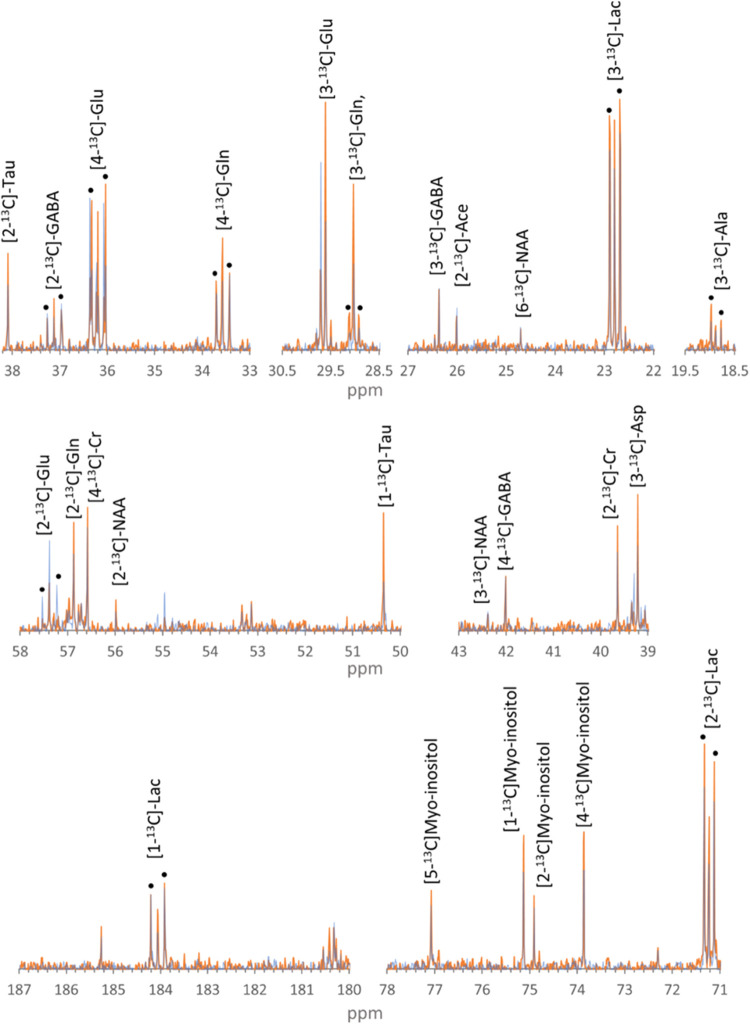
Truncated regions of peaks in mean 13C NMR spectra of aqueous ipsilateral cerebral extracts from [1,2–13C]-glucose infused rodents treated with TNF (orange) and vehicle (blue). Black dots are above doublet peaks corresponding to the labeled singlet, indicating metabolism via glycolysis (doublets) and the pentose phosphate pathway (singlet). Ala, alanine; Ace, acetate; Asp, aspartate; Cr, creatine; GABA, γ-aminobutyric acid; Gln, glutamine; Glu, glutamate; Lac, lactate; NAA, N-acetyl aspartate; and Tau, taurine.

To investigate the impact of TNF-induced metabolite changes in glia and neurons in further detail, rodents were infused with [1,2-^13^C]-glucose. This stable isotope labeled substrate allows glycolysis, PPP, TCA cycle, PDH, and PC metabolism to be simultaneously probed in whole brain tissue extracts via *ex vivo* NMR. Metabolites detectable in ^13^C NMR spectra of aqueous brain extracts of rats infused with [1,2-^13^C]-glucose included alanine, lactate, NAA, acetate, GABA, glutamine, glutamate, taurine, aspartate, creatine, and myo-inositol (Figure 4).

**Figure 5:**
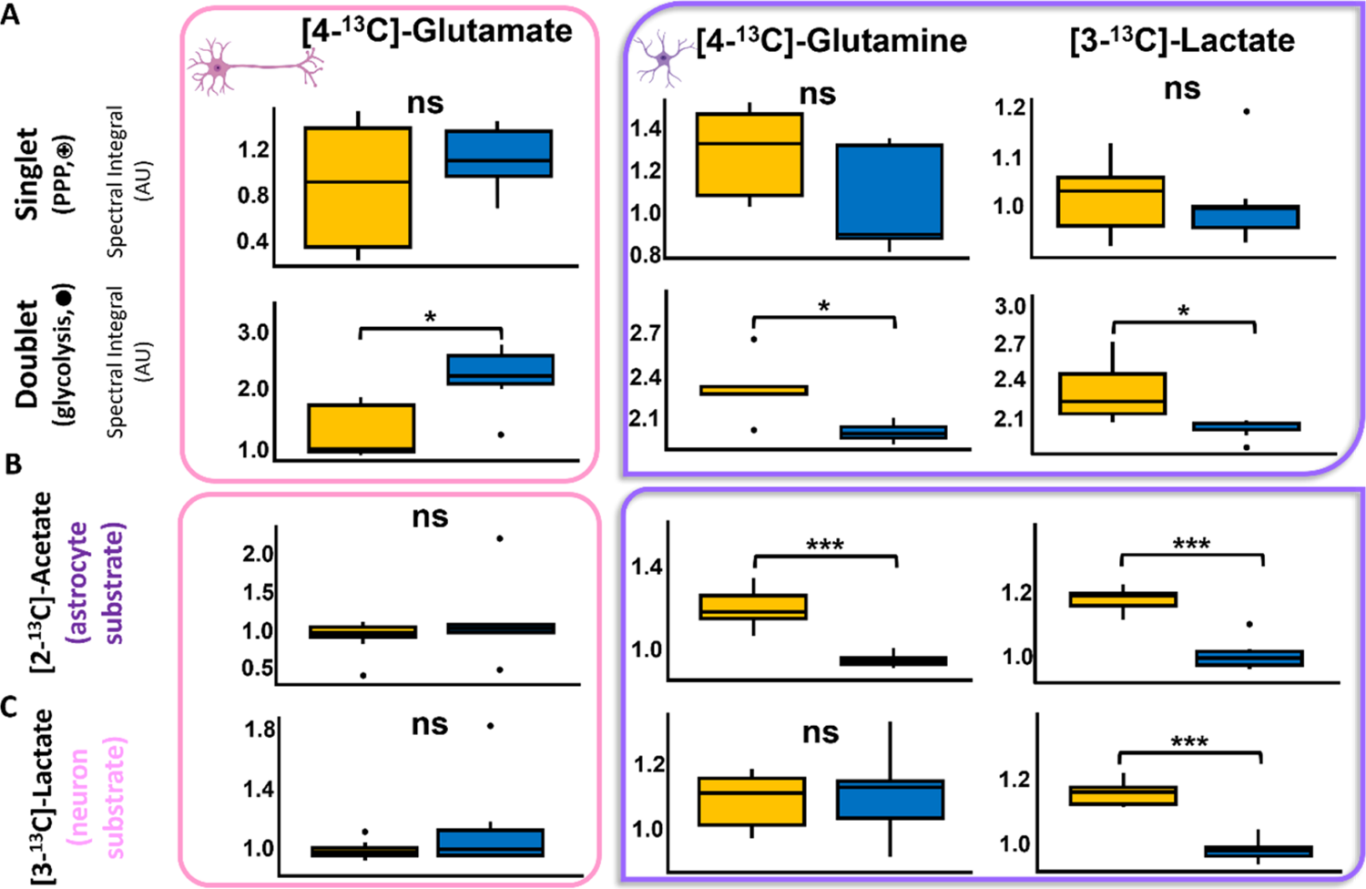
Box plots of 13C labeled metabolites produced via astrocytic (purple) and neuronal (pink) pathways. A. Singlet and doublet peaks for TNF (yellow) and vehicle-treated control (blue) metabolites derived from [1,2–13C]-glucose. B. Doublet only peaks of metabolites derived from [2–13C]-acetate (astrocytic substrate) and C. [3–13C]-lactate (neuronal substrate). Student’s *t* test *p*-values less than 0.05, 0.01, and 0.001 are represented by *, **, ***, respectively. ns; nonsignificant.

A targeted approach was applied to the ^13^C spectra to probe glycolysis, TCA cycle, and the glutamine/glutamate shuttle in astrocytic and neuronal metabolism.^17^ Therefore, the analysis was limited to peaks corresponding to lactate, glutamine, and glutamate. No significant changes were observed in singlet concentrations, suggesting that no detectable impact of TNF on the PPP flux was observed. On the other hand, doublets reflect the presence of isotopomers with two adjacent ^13^C nuclei, arising from the metabolism of [1,2-^13^C]-glucose to [2,3-^13^C]-pyruvate via glycolysis. Therefore, the significant increase in the relative concentrations of doublet resonances of [4-^13^C]-glutamine, [3-^13^C]-lactate, and decrease in [4-^13^C]-glutamate suggest that TNF significantly alters glycolytic metabolic flux resulting in changes in these downstream metabolites (Figure 5A). It should be noted that no significant difference in total glucose concentration was observed in either vehicle control or TNF-treated brain extracts, confirming that changes in ^13^C labeling patterns are due to changes in glycolytic flux and not changes in the total pool of glucose.

The ^13^C labeling of glutamate and glutamine at the C4 position arises from pyruvate metabolism via PDH. Given that glutamine is produced from glutamate via an astrocyte-specific enzyme, GS, the significant increase in [4,5-^13^C]-glutamine likely reflects increased astrocytic activity.^37^ This is supported by previous publications which have shown that TNF increases glutaminase activity, catalyzing glutamate production from glutamine.^38^

TNF treatment also resulted in significant increases in doubly labeled C2 and C3 lactate isotopomers as a result of glycolysis either in the brain or blood. Lactate is considered an alternative energy substrate,^16−18^ which can be metabolized to produce glutamate in the TCA cycle for use as a neurotransmitter, suggesting that relative changes in lactate should also be reflected in glutamate. Thus, the increased levels of [2,3-^13^C]-lactate seem incongruous with the decreased levels of [4,5-^13^C]-glutamate. Instead, coupled with the increase in [4,5-^13^C]-glutamine, these results lead to the hypothesis that increased astrocytic activity following TNF treatment leading to the production of glutamine and lactate but decreased neuronal capacity to utilize lactate and glutamine to produce glutamate. A similar phenomenon has been previously observed in brain trauma.^39^

In order to confirm that the observed changes in [1,2-^13^C]-glucose-infused animals were indeed due to upregulation of astrocytic metabolism, the experiments were repeated with [2-^13^C]-acetate. The TNF-induced increases in astrocyte-derived [4,5-^13^C]-glutamine and [2,3-^13^C]-lactate (resulting from pyruvate recycling in acetate treated astrocytes^40,41^) were confirmed. (Figure 5B). Next, we investigated whether the observed decrease in [4,5-^13^C]-glutamate could be ameliorated by providing an additional alternative substrate for glutamate synthesis. No significant increase in brain glutamate was observed following the peripheral infusion with L-lactate (Figure S6). We confirmed uptake of peripherally infused lactate in brain extracts using [3-^13^C]-lactate; while significant elevation of [3-^13^C]-lactate was observed confirming uptake of lactate in the CNS, there was no corresponding increase in glutamate (Figure 5C) confirming a decreased capacity to utilize lactate as an energy substrate in the TNF-treated brain. Although these increased levels of [3-^13^C]-lactate may be a result of increased lactate uptake resulting in a larger proportion of labeled lactate in TNF-treated brains, metabolism of lactate in the periphery to glucose prior to astrocytic uptake and conversion to lactate cannot be ruled out.

## Conclusions

TNF is known to play a substantial role in neuroinflammation; however, its precise effect on astrocyte and neuronal biochemistry has, hitherto, remained unclear. ^13^C stable isotope tracing by *ex vivo* NMR spectroscopy is a powerful technique that allows several metabolic pathways (glycosolysis, PPP, TCA cycle, and glutamine/glutamate shuttle) to be probed in both glia and neurons in whole tissue extracts simultaneously. Here, we demonstrate that elevated TNF levels have a profound impact on the NMR-detectable brain metabolome (^1^H NMR spectrum) while the metabolic network is rearranged (^13^C tracer experiments). TNF is known to generate reactive astrocytes.^27^ Consistent with the elevated levels of astrocyte-derived glutamine and lactate observed here in conjunction with the decreased glutamate concentrations point to a decrease in neuronal capacity to utilize these metabolites, which may promote the neurotoxic effects of TNF. This disruption of the glutamine/glutamate and lactate shuttles suggests that activated astrocytes prioritize their behavior to generate, for instance, glial scars and absent themselves from their key role to support neuronal function. While a reduction of neuronal support in favor of scar formation may be the lesser of two evils, there is no doubt that an excessive astrocyte response, in this way, would contribute to the death of neurons. Thus, TNF-mediated changes in brain metabolic fluxes could contribute to the pathophysiology of diseases like Alzheimer’s, Parkinson’s, and multiple sclerosis, where neuroinflammation is a key feature.

## Supporting Information Available

Figure S 1. Percentage enrichment in the ^13^C-infused brain tissue; Figure S 2. PCA scores plot for ^1^H NMR spectra showing clear separation between samples intracranially injected with TNF-α or vehicle with intravenous [1,2-^13^C]-glucose infusion; Figure S 3. GFAP Immunohistochemistry; Figure S 4. PCA loadings plot; Figure S 5. Box plots illustrating significant changes in the ipsilateral/contralateral hemisphere ratios of ^1^H-detecable metabolites; Figure S 6. 4,5-^13^C glutamate levels following L-lactate and [1,2-^13^C]-glucose infusion in vehicle-treated (saline) and TNF-treated brain extracts. Table S 1; Assigned ^1^H spectral bins (PDF)

## Abbreviations

CoA: coenzyme A
AMP: adenosine monophosphate
ATP: adenosine triphosphate
G6P: glyceraldehyde-6-phosphate
GS: glutamine synthetase
PC: pyruvate carboxylase
PCA: Principal component analysis
PDH: pyruvate dehydrogenase
PPP: pentose phosphate pathway
TNF: tumor necrosis factor α
