# Supplementary material for: Ex-Vivo 13C NMR Spectroscopy of Rodent Brain: TNF Restricts Neuronal Utilization of Astrocyte-Derived Metabolites
Source: J Proteome Res. 2024 Jun 29;23(8):3383–92. doi: 10.1021/acs.jproteome.4c00035 (PMC11301676; doi:10.1021/acs.jproteome.4c00035)
Supplement: Supplementary file 1 — pr4c00035_si_001.pdf [file pr4c00035_si_001.pdf]

Supplementary Info

Ex-Vivo ^{13}C NMR Spectroscopy of Rodent Brain: TNF Restricts Neuronal Utilisation of Astrocyte-Derived Metabolites

Daniel Radford-Smith[1,2,‡], Tang T. Ng[1,‡], Abi G. Yates[1,2,†], Isobel Dunstan[2], Timothy D.W. Claridge[1,±], Daniel C. Anthony[2], and **Fay Probert**[1*]

1. Department of Chemistry, University of Oxford, Oxford, OX1 3TA, UK.

2. Pharmacology Department, University of Oxford, Oxford, OX1 3QT, UK

Corresponding Author

*Fay Probert. E-mail: Fay.probert@chem.ox.ac.uk. Tel: +44 1865 275713

Present Addresses

†Icahn School of Medicine at Mount Sinai, New York, NY, USA

±Exscientia, The Schrödinger Building, Oxford Science Park, Oxford OX4 4GE, United Kingdom

KEYWORDS Ex vivo NMR, stable isotope tracing, neuroinflammation, metabolomics, astrocyte, tumor necrosis factor.

Table of Contents:

Figure S 1. Percentage enrichment in ^{13}C infused brain tissue.

Figure S 2. PCA scores plot for ^{1}H NMR spectra showing clear separation between samples intracranially injected with TNF-α or vehicle with intravenous [1,2-^{13}C]-glucose infusion.

Figure S 3. GFAP Immunohistochemistry.

Figure S 4. PCA loadings plot.

Figure S 5. Box plots illustrating significant changes in the ipsilateral/contralateral hemisphere ratios of ^{1}H-detecable metabolites.

Figure S 6. 4,5-^{13}C glutamate levels following L-lactate and [1,2-^{13}C]-glucose infusion in vehicle treated (saline) and TNF treated brain extracts.

Table S 1. Assigned ^{1}H spectral bins.

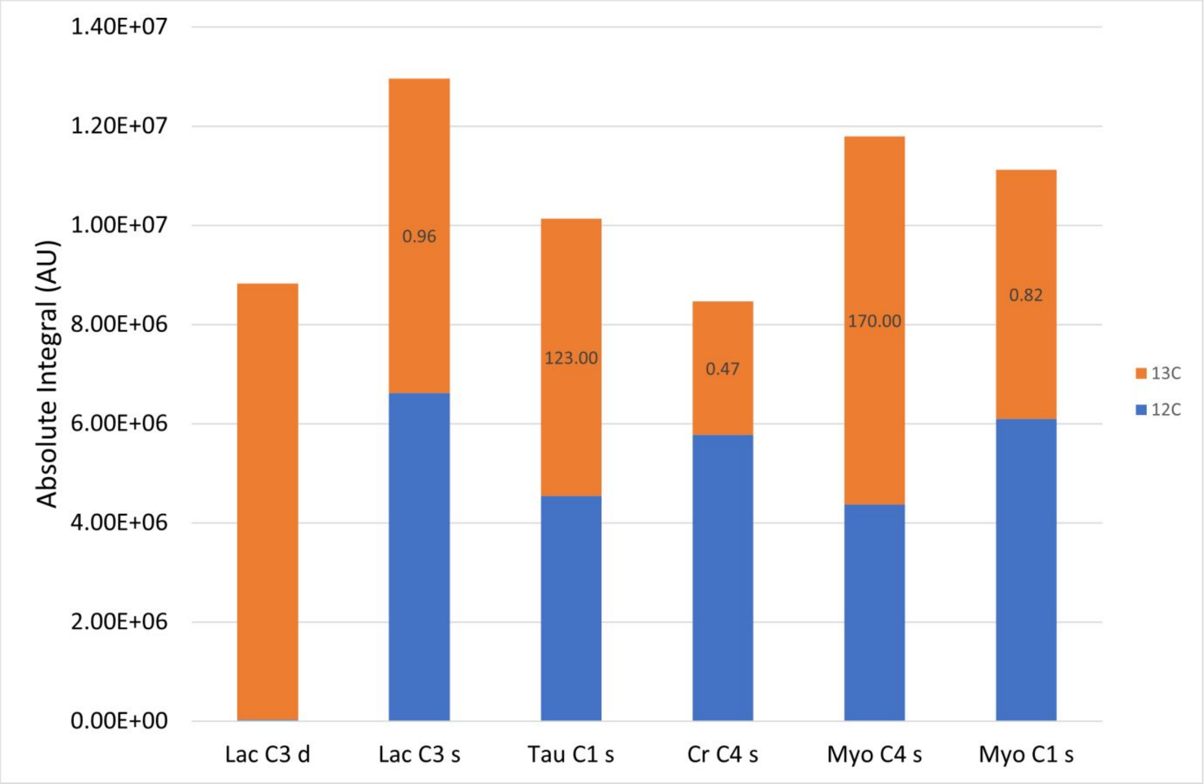

Figure S 1. Percentage label incorporation in [1,2-13C]-glucose infused brains relative to natural abundance 12C-glucose infused for key metabolites.

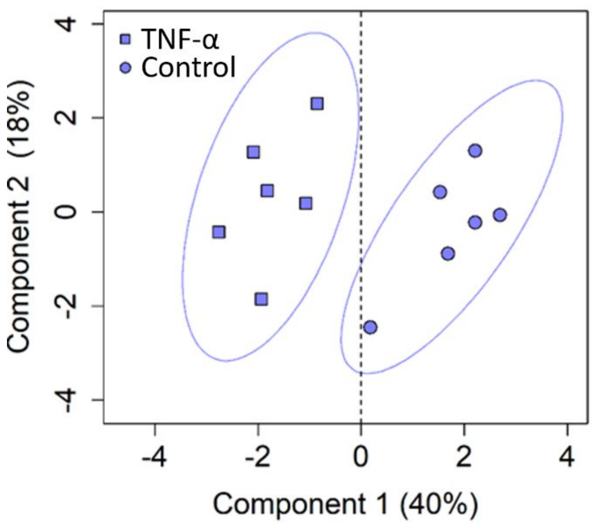

Figure S 2. PCA scores plot for ^{1}H NMR spectra showing clear separation between samples intracranially injected with TNF-α or vehicle with intravenous [1,2-^{13}C]-glucose infusion. R2 = 0.596 indicating the model accounts for a good proportion of variation and fits the data well.

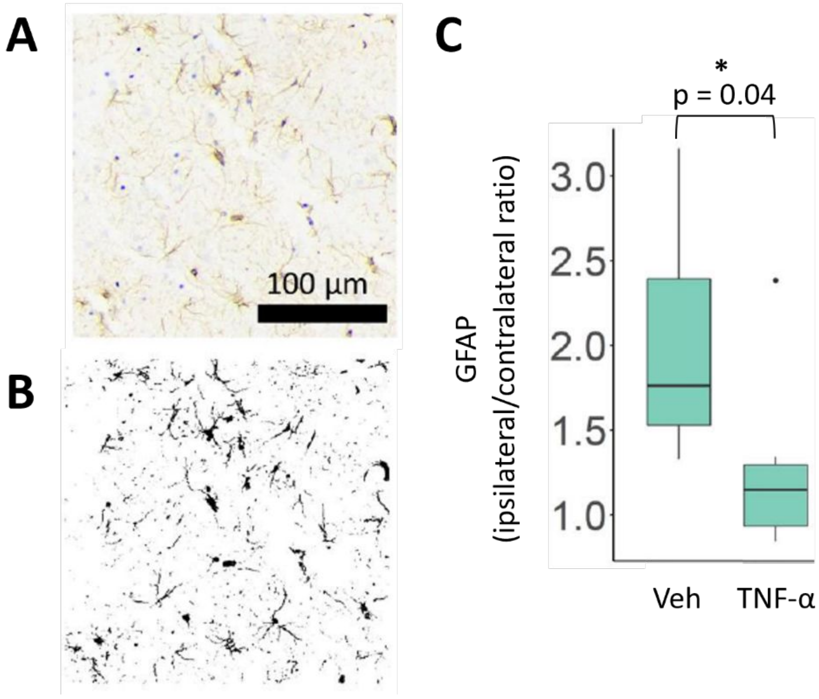

Figure S 3. GFAP Immunohistochemistry. Representative images A) of GFAP stained astrocytes and B) used for quantification following applied colour threshold. C) Box plot showing normalised area of staining of vehicle (Veh) and TNF-α treated brain.

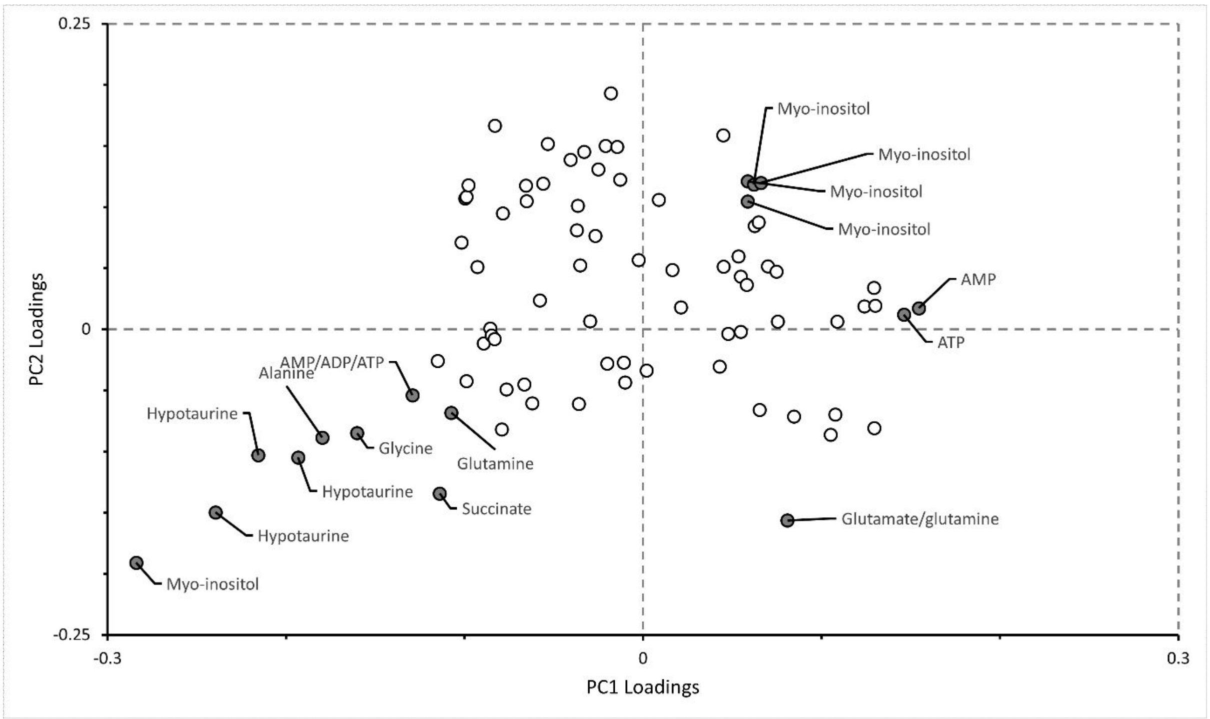

Figure S 4. PCA loadings plot (R2 = 0.584) associated with scores plot in Figure 3 which shows separation between TNF and vehicle-treated control groups following intravenous infusion with [2-13C]-acetate, [1,2-13C]-glucose, or [3-13C]-lactate. Variables which changed significantly as a result of TNF treatment are labelled.

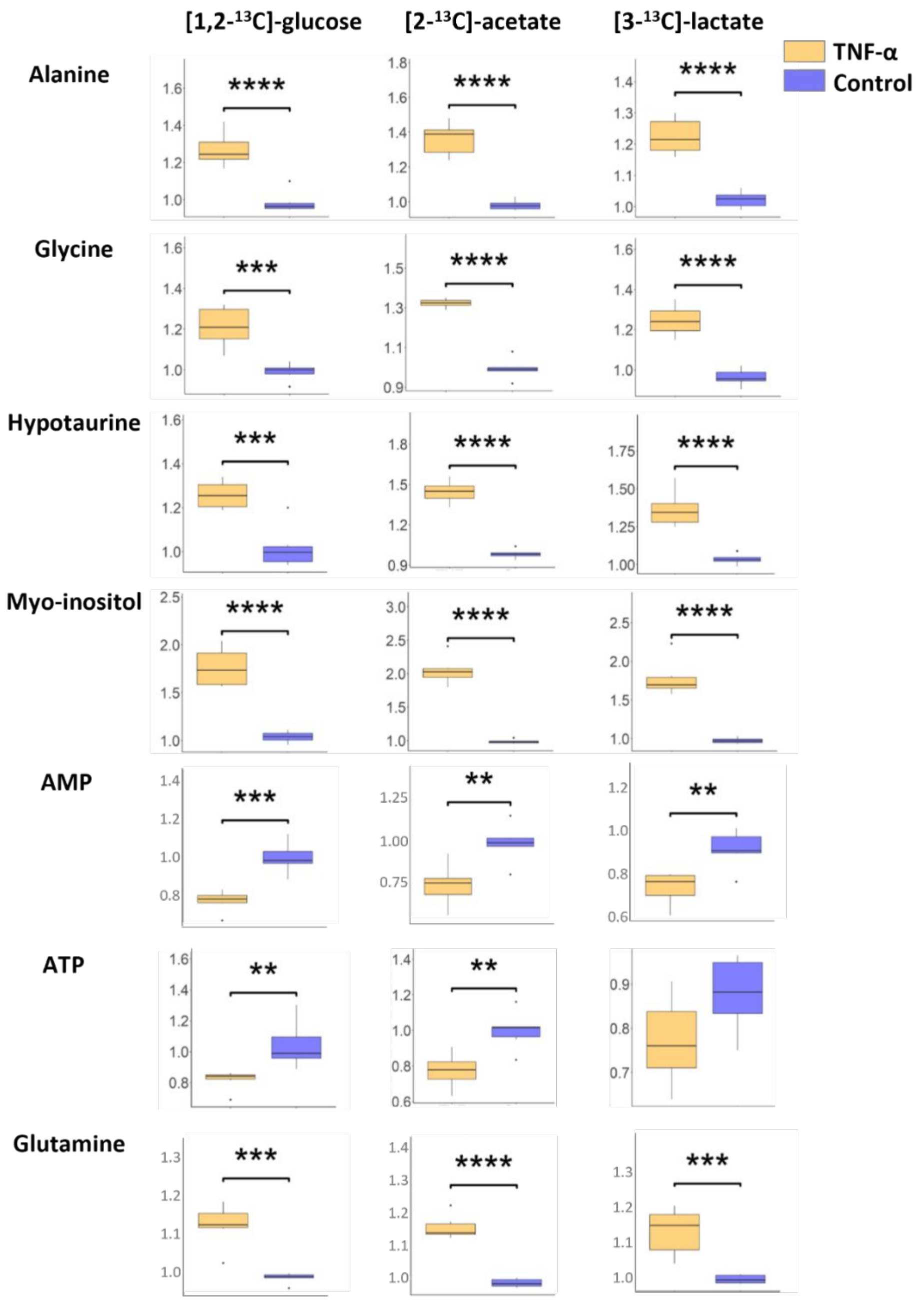

Figure S 5. Box plots illustrating significant changes in the ipsilateral/contralateral hemisphere ratios of ^{1}H-detecable metabolites in TNF-α treated (yellow) and vehicle control (blue) brain extracts following infusion with either [1,2-^{13}C]-glucose, [2-^{13}C]-acetate, or [3-^{13}C]-lactate. Student's t-test p-values less than 0.05, 0.01, 0.001, and 0.0001 are represented by *, **, ***, and **** respectively.

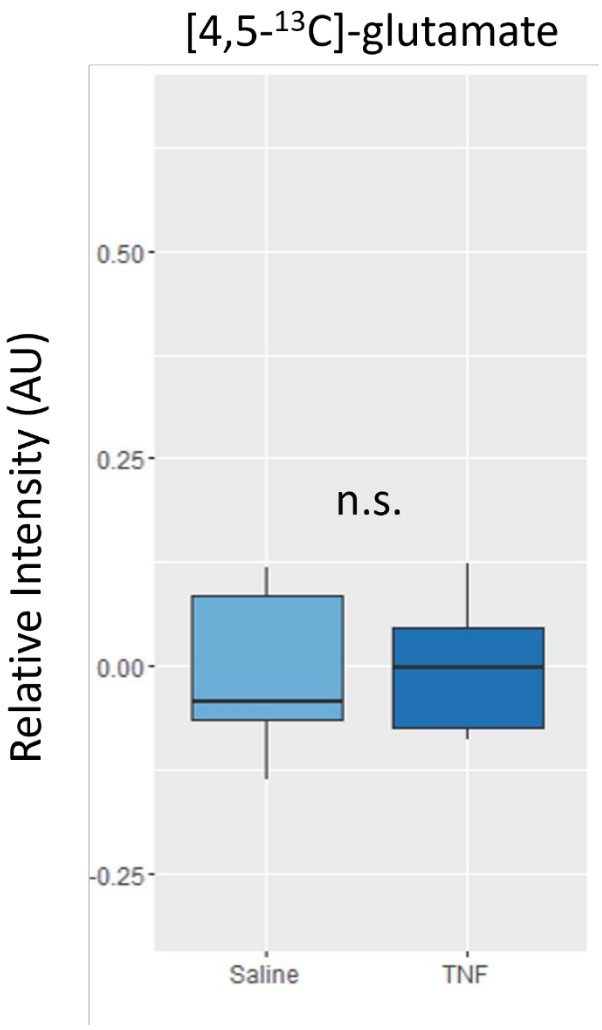

Figure S 6. 4,5-^{13}C glutamate levels following L-lactate and [1,2-^{13}C]-glucose infusion in vehicle treated (saline) and TNF treated brain extracts. Despite infusion if lactate no significant change in glutamate production was observed. n.s.; non-significant.

Table S1. Assigned ^{1}H spectral bins. Overlapping assignments within a binned region are represented by /.

Metabolite	J / Hz		ppm
Acetate	s		[1.92 .. 1.92]
Adenosine	s		[8.23 .. 8.24]
	s		[8.34 .. 8.35]
Alanine	d	7.3	[1.48 .. 1.49]
AMP/ADP/ATP	s		[8.27 .. 8.27]
	s		[8.27 .. 8.28]
ADP/ATP	s		[8.53 .. 8.55]
AMP	s		[8.61 .. 8.62]
ATP	d	5.8	[6.14 .. 6.14] , [6.14 .. 6.15]
Ascorbate	d	1.9	[4.51 .. 4.52]
Aspartate	dd	8.7, 17.5	[2.66 .. 2.66], [2.67 .. 2.67], [2.70 .. 2.70]
	dd	3.7, 8.9	[3.89 .. 3.91]
Choline	s		[3.20 .. 3.21]
Creatine	s		[3.04 .. 3.04]
	s		[3.93 .. 3.94]
GABA	t	7.4	[2.29 .. 2.31]
	t	7.5	[3.00 .. 3.03]
Glutamate	m		[2.04 .. 2.09]
	m		[2.34 .. 2.37]
Glutamine	m		[2.10 .. 2.16]
	m		[2.44 .. 2.47]
Glutamate/glutamine	m		[3.73 .. 3.74], [3.74 .. 3.78], [3.79 .. 3.79]
Glycine	s		[3.56 .. 3.56]
Hypotaurine	t	6.7	[2.64 .. 2.64] , [2.65 .. 2.65]
	t	6.9	[3.35 .. 3.37]
Isoleucine	d	7.2	[1.02 .. 1.02]
Lactate	d	6.9	[1.32 .. 1.34]
	q	7.0	[4.09 .. 4.13]

LDL (Low Density Lipoprotein)	broad		[0.86 .. 0.89]
Myo-inositol	t	9.5	[3.27 .. 3.27], [3.28 .. 3.28]
	dd	2.9, 10.0	[3.52 .. 3.55]
	t	9.6	[3.61 .. 3.64]
	t	2.9	[4.06 .. 4.07]
NAA	s		[2.02 .. 2.02]
	dd		[2.48 .. 2.48] , [2.49 .. 2.52]
	dd		[2.68 .. 2.68] , [2.70 .. 2.71]
	dd	3.7, 10	[4.38 .. 4.39] , [4.40 .. 4.41]
NAA + Aspartate			[2.68 .. 2.69]
NAA + ?			[2.79 .. 2.84]
NAD^+	d	5.8	[6.10 .. 6.10] , [6.10 .. 6.11]
	s		[8.43 .. 8.43]
	s		[9.34 .. 9.35]
NADH	d	5.4	[6.15 .. 6.16]
	s		[8.21 .. 8.22]
NAG (N-Acetylglutamate)	m		[1.88 .. 1.91]
Phosphatidylcholine (GPCho)	s		[3.23 .. 3.23]
Phosphocholine	s		[3.22 .. 3.23]
Pyroglutamate	m		[2.42 .. 2.43]
	m		[2.55 .. 2.57]
Scyllo-inositol	s		[3.35 .. 3.35]
Succinate	s		[2.40 .. 2.41]
Taurine	t	6.6	[3.25 .. 3.27] , [3.27 .. 3.28]
	t	6.7	[3.41 .. 3.43]
Tyrosine	d	8.5	[7.19 .. 7.20]
Valine	d	6.9	[0.99 .. 1.00]
	d	7	[1.04 .. 1.05]
